# Ultralow Quiescent Power‐Consumption Wake‐Up Technology Based on the Bionic Triboelectric Nanogenerator

**DOI:** 10.1002/advs.202000254

**Published:** 2020-05-11

**Authors:** Chenxi Zhang, Keren Dai, Di Liu, Fang Yi, Xiaofeng Wang, Lianqing Zhu, Zheng You

**Affiliations:** ^1^ Department of Precision Instrument Tsinghua University Beijing 100084 P. R. China; ^2^ ZNDY of Ministerial Key Laboratory School of Mechanical Engineering Nanjing University of Science and Technology Nanjing Jiangsu 210094 P. R. China; ^3^ Beijing Institute of Nanoenergy and Nanosystems Chinese Academy of Sciences Beijing 100083 P. R. China; ^4^ School of Materials Science and Engineering Sun Yat‐sen University Guangzhou Guangdong 510275 P. R. China; ^5^ Beijing Innovation Center for Future Chips Tsinghua University Beijing 100084 P. R. China; ^6^ Center for Flexible Electronics Technology Tsinghua University Beijing 100084 P. R. China; ^7^ School of Instrument Science and Opto‐Electronic Engineering Beijing Information Science and Technology University Beijing 100192 P. R. China

**Keywords:** bionic triboelectric nanogenerators, low quiescent power consumption, scene judgment, self‐powered sensors, wake‐up systems

## Abstract

Wake‐up circuits in smart microsystems make huge contributions to energy conservation of electronic networks in unmanned areas, which still require higher pressure‐triggering sensitivity and lower power consumption. In this work, a bionic triboelectric nanogenerator (bTENG) is developed to serve as a self‐powered motion sensor in the wake‐up circuit, which captures slight mechanical disturbances and overcomes the drawback of conventional self‐powered motion sensors in the wake‐up circuit that the circuit can only be triggered when a considerable pressure is applied on the sensor. The bTENG mimics the structure of plants and the addition of the leaf‐shaped tentacle structures can increase the electrical outputs by four times, which largely extends the detection range of the wake‐up circuit. The bTENG can detect both noncontact and contact mechanical disturbances; and voltages generated from both situations can trigger the wake‐up system. Moreover, the specially designed circuit that is compatible with the bTENG can help more accurately control the wake‐up system and prolong the battery life of the electronic networks to 12.4 times. An intrusion detection system is established in the wake‐up circuit to distinguish human motion and judge the scene. This work opens new horizons for wake‐up technologies, and provides new routes for persistent sensing.

## Graphical abstract

A wake‐up system with low quiescent power consumption, large wake‐up range, and ability to distinguish false trigger is developed based on a plant‐shaped bionic triboelectric nanogenerator, which can be awakened by contact and noncontact mechanical disturbances. This work provides new routes for persistent sensing and may have broad applications in areas such as environmental monitoring, threat detection, perimeter monitoring, and critical infrastructure protection.

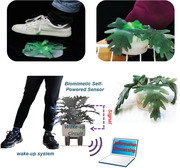

## 1 Introduction

With the advent of the information age, the smart microsystem, which is a combination of micro‐electro‐mechanical system and microelectronics technology, has become a global research hotspot. It integrates sensors, actuators, signal collectors, data processors, and control circuits for applications in unattended security, infrastructure monitoring, and the Internet of things. To enhance the service life of the sensor network in the area where the battery cannot be replaced, the defense advanced research projects agency proposed the concept of “Near Zero Power RF and Sensor Operations (N‐ZERO)” that is, wake‐up systems sense the environment 100% of the time with ultralow quiescent power consumption and use the energy in signals to activate an internal signal processing circuit.^[^ ^1^, ^2^ ^]^ However, the problems faced in the research are as follows. First of all, since most of the common sensors require power supplies, it is a great challenge to reduce quiescent power consumption. Second, the current motion sensors of wake‐up circuits generally require large amplitude vibrations in order to generate large enough voltages to wake up the circuit. Third, the fabrication processes of the existing sensors used in wake‐up systems are generally complex and expensive. To further reduce the quiescent power consumption, one possible solution is to replace the power‐consuming sensors with self‐powered sensors that can work without system power supply; and to increase the wake‐up system's wake‐up range and sensitivity towards slight mechanical action, new sensors that are capable of generating large enough voltages to trigger the wake‐up circuit under slight touch or even no direct touch need to be developed. In addition, sensors with simple and scalable fabrication processes are desirable, from the perspectives of economy and large‐scale production.

At present, there are self‐powered sensors that have been used in the wake‐up circuit for detection of mechanical disturbance, which are mostly piezoelectric nanogenerators (PENGs).^[^ ^3^, ^4^, ^5^ ^]^ However, the PENGs, which work based on the piezoelectric effect, have low output power density and strict applied conditions. The PENGs can only generate electrical outputs when an applied pressure induces strain inside the piezoelectric material, and the small voltage generated by a slight touch cannot easily overcome the high switching threshold in the wake‐up circuit. These characteristics impose limits on the sensing ability of slight touch and wake‐up range of the wake‐up system. Therefore, low power‐consumption wake‐up systems with self‐powered motion sensors that can sense weak mechanical disturbances and have long sensing distances are in urgent demand. The triboelectric nanogenerator (TENG), which is based on the conjunction of contact electrification and electrostatic induction,^[^ ^6^, ^7^, ^8^ ^]^ has attracted intense attention since its invention.^[^ ^9^, ^10^, ^11^, ^12^, ^13^, ^14^, ^15^, ^16^, ^17^, ^18^, ^19^, ^20^, ^21^, ^22^, ^23^, ^24^ ^]^ Since the TENG has the property of zero power consumption and can output high voltages even when triggered by noncontact mechanical movements, the TENG is a possible solution to meet the demands of self‐powered sensors for wake‐up systems that may enable the system to wake up under slight touch and enlarge the wake‐up range. In addition, the TENG has the advantages including low cost, lightweight property and simple fabrication.

In this work, a plant‐shaped bionic TENG with no power consumption is developed in the wake‐up system, which can trigger the wake‐up system under slight touch or even no touch and largely extends the battery life of the system. Compared with the PENGs that are commonly used in the wake‐up circuits, the bTENG is superior for slight touch sensing. Moreover, the addition of leaf‐shaped tentacles and nanostructures in the bTENG increases the output voltage by four times when sensing slight touch, which improves the detection range of the wake‐up system in the process of environmental monitoring. Also, a wake‐up circuit is specially designed to be compatible with the bTENG and achieve ultralow quiescent power consumption. The wake‐up system with the bTENG can extend the battery life of the electronic network to 12.4 times. To highlight the characteristics of intelligence in our system, a computer program is elaborated to distinguish human motion and judge the wake‐up scene according to the audio signal recorded by the microcontroller after awaking. This work endows the TENG with a new role as a self‐powered sensor for slight touch in the wake‐up circuit and opens up new ideas for the design of wake‐up systems with ultralow quiescent power consumption.

## 2 Results and Discussion

### 2.1 Overview of the bTENG and Wake‐Up System

**Figure 1:**
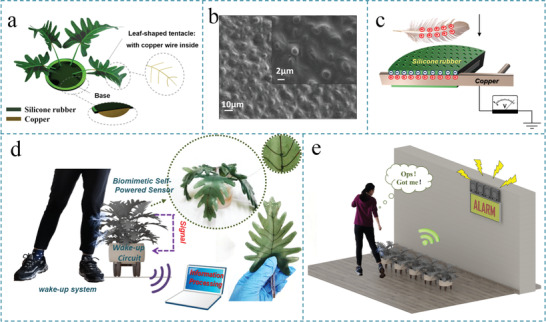
Overview of the bTENG and wake‐up system. a) Schematic diagram showing the structure of the plant‐shaped bTENG. b) SEM images showing the micro/nanostructures on the bTENG leaves’ surface. c) Schematic illustrations of the operation mechanism for the TENG. d) Schematic diagrams showing the bTENG as a self‐powered sensor for wake‐up circuits and the photographs of the bTENG. e) Schematic diagrams of the bTENGs in unattended security that will activate the alarm system when intruders enter the core area.

The typical structure of the bTENG is illustrated in **Figure** **1**a, which consists of a base plane and plenty of flat leaf‐shaped tentacles. The base is composed of an internal metal plate as the electrode and an external silicone rubber layer as the dielectric layer. Plenty of flat leaf‐shaped tentacles are attached to the internal metal plate, which enlarge the contact area in three‐dimensional space and lead to higher electrical outputs. The leaf‐shaped tentacle is composed of an internal vein‐like metal wire and an external leaf‐shaped silicone rubber layer. The vein‐like wires endow the bTENG with desirable mechanical properties such as high mechanical strength, hardness, and plasticity.

The micro/nanostructures imitating the nanostructures of lotic leaves (Figure 1b) are created on the silicone rubber surface to increase the contact surface area using a modeling method. Note that the detailed fabrication process of the surface nanostructures can be found in the Experimental Section and Figure S1, Supporting Information.

The silicone rubber usually becomes negatively charged after contact with another material because of its strong ability to attract electrons and the triboelectric charges on the silicone rubber's surface can be maintained for a long period of time.^[^ ^25^, ^26^ ^]^ The periodical contact and separation between the single‐electrode‐mode bTENG and the approaching object (feather, fur, etc.) can causes electrical potential variation between the copper electrode and the ground in the open‐circuit condition (Figure 1c).^[^ ^20^, ^27^ ^]^ There will also be generated electrical outputs when the moving object do not directly touch the bTENG because of the electrostatic effect.

This bTENG is a trump card for capturing slight external mechanical disturbances with the characteristic of no power consumption. Based on these features, the bTENG is applied to the wake‐up system suitable for the bTENG innovatively as a slight touch sensor, which is the core technology to monitor unmanned areas. When an intruder passing by, the system can wake up, process the collected sound, and judge the safety of the scene (Figure 1d). Besides, the bTENG's unique structure enhances its concealment in natural environment and makes it more useful in myriad unmanned monitoring fields such as automated factory detections, intelligent home systems, and border inspections (Figure 1e).

### 2.2 Optimized Structure Design of the bTENG

**Figure 2:**
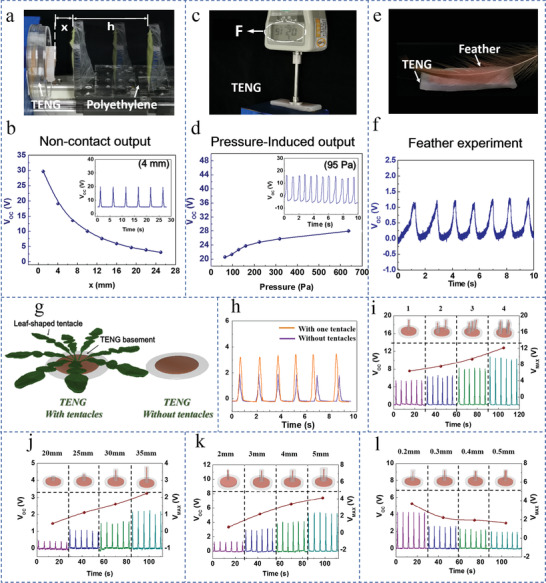
Detection of slight mechanical disturbances and structure optimization of the bTENG. a) Photograph showing the experimental setup for the noncontact experiment. b) The relationship between the output voltage of the TENG and the nearest noncontact distance. The inset shows the output voltage of the TENG when the nearest noncontact distance is 4 mm. c) Photograph is showing the experimental setup for the contact experiment. d) The relationship between the output voltage of the TENG and the exerted pressure during contact. The inset shows the output voltage of the TENG when exerted by a pressure of 95 Pa. e) Photograph showing the experimental setup for the feather‐touch experiment. f) The output voltage of the TENG in the feather‐touch experiment. g) Schematic diagrams of the TENG with/without tentacles. h) Effects of the TENG's tentacles structure on output voltage. i) The relationship between the output voltage of the TENG and the number of tentacles. j) The relationship between the output voltage and the length of tentacles. k) The relationship between the output voltage and the layer thickness of the external silicone rubber tentacles. l) The relationship between the output voltage and the radius of the internal wire tentacles.

The TENG exhibits excellent performance for the detection of noncontact and slight‐touch external mechanical disturbances. Note that the term slight touch discussed in this article is that the two surfaces come into contact and the applied pressure during contact is near zero. In the experiments, we divide the external mechanical motion into two types: noncontact motion and contact motion that exerts pressure during contact. For the experimental setup in the testing of the noncontact motion, the TENG was fixed in a slot and was approached by a polyester that was attached to a working plate and driven by a stepping motor at a uniform speed (**Figure** **2**a; Movie S1, Supporting Information). The relationship between the nearest distance from the TENG and the TENG's output voltage is recorded (Figure 2b). It is found that the output voltage of the bTENG decreases with the increasing nearest distance of the moving object from the bTENG. When the nearest distance is 4 mm, the TENG can produce a voltage of up to 20 V, which demonstrates TENGs’ sensing ability of noncontact motion.

In the experiment of contact motion that exerts pressure during contact, we used a gauge to control the force applied on the sensors’ surface (Figure 2c). The applied pressure is obtained by dividing the applied force by the contact area and the relationship between the output voltage and the pressure is recorded (Figure 2d). In this case, the pressure sensitivity is 34.4 mV Pa^–1^ when the pressure is less than 160 Pa and the pressure sensitivity is 8.3 mV Pa^–1^ when the pressure is higher than 160 Pa (Figure S2, Supporting Information). The TENG produces continuous pulse voltage signals of 23 V when exerted by a pressure of 95 Pa. Moreover, it is found that when objects made of different materials exert pressure on the TENG, the maximum output voltages are all more than 6 V when the pressure is less than 700 Pa (Figure S3, Supporting Information). These two experiments demonstrate TENG's superiority as a self‐powered sensor for detecting noncontact and slight touch disturbances.

To further confirm the TENG's superior performance in detecting slight touch, we compared the performance of the TENG with that of the PENG, which is the most commonly used sensor in low‐power wake‐up circuits at present.^[^ ^28^, ^29^, ^30^ ^]^ It is found that the electrical output of the TENG is 20 times higher than that of the PENG in the slight‐touch experiment; and the PENG generates no electrical outputs in the noncontact experiment (Figure S4a,b, Supporting Information). Note that the detailed theoretical explanation for the TENG's superior performance to the PENG's in slight‐touch sensing can be found in Note S1, Figures S4c and S5, Supporting Information. A feather‐touch experiment is also conducted to demonstrate the TENG's advantage in detecting slight touch. When a feather wavered to the bTENG, the bTENG generates a voltage of 1.2 V (Figure 2e,f; Movie S2, Supporting Information). However, the PENG generates no electrical outputs when a feather wavered to the PENG (Figure S4d, Supporting Information). It can be seen from the above experiments that TENGs are superior in slight touch detections for wake‐up systems.

To further enhance the output voltage of the bTENG and thus extend the detection range towards slight mechanical disturbances of the wake‐up circuit, we add many blade‐shaped tentacles to the basic flat‐shaped bTENG (Figure 2g). We first simplified the shape of the tentacles into cylindrical structures (Figure S6a, Supporting Information) to investigate the effect of the tentacle on the output voltage. bTENGs with and without a tentacle were placed on the experimental setup and approached by the same material, respectively. The output voltage signals show that the addition of just one tentacle structure can double the output voltage (Figure 2h). The simulation results also prove the feasibility of the bTENG with the tentacle structure (Figure S6b,c, Supporting Information). Note that the cylindrical tentacle applied in the experiment is to investigate the general impact of the device's structure parameters on the electrical outputs; and the shape of the tentacle could also influence the electrical outputs, that is, larger surface area can accumulate more triboelectric charges and thereby results in higher electrical outputs.

The effects of the number of the tentacle (Figure 2i), length of the tentacle (Figure 2j), layer thickness of the external silicone rubber (Figure 2k), and radius of the internal wire (Figure 2l) on the output voltage are studied systematically. The experiments indicate that the addition of more and longer tentacles with thicker external silicone rubber and finer internal wire can increase the output voltage of the bTENG. Note that the simulations about the effects of parameters of device structure on the electrical outputs have also been done, which have the same trend as the experimental results (Figure S7, Supporting Information). The simulation results also prove that the uniform distribution of tentacles is also conducive to enhance the output voltage (Figure S8, Supporting Information). Basic characteristics of the tentacles in the bTENG (large leaf surface area, long branch length, a large number of leaves, fine veins, uniform distribution) conform to the above requirements. The study on the shape of tentacles lays the foundation for our bionic design theory. At the same time, the bTENG has the advantages of simple fabrication process, low cost, high repeatability, and strong plasticity. The bTENGs also can imitate different species of plants according to various application scenarios.

### 2.3 Design of the Wake‐Up Circuit

**Figure 3:**
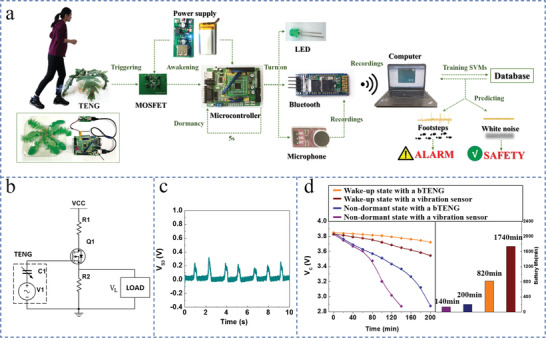
The design of wake‐up circuits using bTENGs as sensors. a) Schematic diagrams showing the wake‐up circuit's working principle. b) Circuit diagram of the switch element MOSFET connected to the bTENG. c) The output voltage of the switching element triggered by the bTENG. d) Comparison of the power consumptions of the wake‐up systems and nondormant systems.

Since the bTENG has the property of zero power consumption and can output high voltages even when triggered by noncontact mechanical movements, it can be applied in the wake‐up system as a self‐powered sensor. A wake‐up circuit suitable for the bTENG that is composed of a switch component and a data processing microcontroller has been designed, which together with the bTENG form the wake‐up system (**Figure** **3**a). Considering that the bTENG has the characteristics of high voltage output and low current output, and that the output voltage of the TENG under load is impacted by the load impedance, a metal‐oxide‐semiconductor field‐effect transistor (MOSFET) is attached to the bTENG as a switch element (Figure 3b), which has the characteristics of easy conduction, no restriction on the input current, and low power consumption.^[^ ^31^, ^32^ ^]^ Since the actual output of the bTENG in a circuit will be influenced by the load impedance, the wake‐up system can be more accurately controlled by connecting the bTENG to the gate electrode of the MOSFET with a known impedance.

Two parameters should be paid attention to when choosing the MOSFET suitable for the bTENG: the threshold voltage and the parasitic capacitance. The input voltage controls the charge carrier channel between the source and drain electrodes in a MOSFET. When the input voltage is higher than the threshold voltage of the MOSFET, the switch will turn on and give a trigger voltage to the microcontroller to start the whole circuit, which indicates that we can control the wake‐up voltage of the wake‐up system by selecting a MOSFET with a certain threshold voltage. When the bTENG is connected with the MOSFET, the parasitic capacitance of the MOSFET represents the load impedance of the bTENG, which is the input capacitance minus the reverse transmission capacitance in the datasheet. To study how the parasitic capacitance of the MOSFET affects the wake‐up system, two types of MOSFET with different parasitic capacitances (PMZ600UNEL and IRF1404) are connected to the bTENG, respectively. It is found that when the open‐circuit voltages of the bTENG are the same, the smaller the MOSFET's parasitic capacitance, the larger the actual output voltage of the bTENG (Figure S9, Supporting Information). Therefore, for practical applications, the bTENG based wake‐up system can be more precisely controlled by adding a MOSFET with a certain threshold voltage and a certain parasitic capacitance according to practical requirements. In addition, when choosing a MOSFET for the wake‐up system, it should be kept in mind that the MOSFET needs to have the characteristic of low drain leakage current in order to reduce the power consumption of the system and high breakdown voltage in order to ensure the safety of the circuit.

In this article, when the voltage signal generated by bTENG turns on the MOSFET, the MOSFET will send a pulse signal to the connected dormant microcontroller and wake up the CPU with short delay (<10 µs) to start the subsequent work. The output signal of the MOSFET with a low threshold voltage (0.45 V) when triggered by the bTENG is illustrated in Figure 3c. Note that the detailed theoretical foundation of the role that the MOSFET plays in the bTENG based wake‐up system can be found in Note S2, Supporting Information.

To distinguish human motion and rule out false triggers, an intrusion detection system is also developed for the scenario that an alarm alert is released only when human intrusion happens. The intrusion detection system is established by analyzing the sound recorded by the peripheral recording device of the microcontroller when unexpected event happens. The specific implementation method is as follows. The triggering signal wakes up the microcontroller, and the microcontroller turns on the recorder to record the audio signal of the wake‐up scene. Then the sound signal is sent to the remote computer through peripheral Bluetooth.^[^ ^33^, ^34^ ^]^ The detailed explanation of the basic description of MSP430 can be found in Note S3, Supporting Information. Using MATLAB software, we write a program extracting the mel‐frequency cepstrum coefficient^[^ ^35^, ^36^ ^]^ (MFCC) of the recorded signal to facilitate the subsequent comparative analysis which greatly reduce data quantity by 2%. Methods of extracting the MFCC and the key code can be found in Note S4, Supporting Information. After learning 50 groups of characteristic parameters of environmental noise and ambient sounds (human footsteps) by support vector machine learning method,^[^ ^37^, ^38^ ^]^ which can be changed according to the factual requests, the program can compare the received recording with the previous learning result and judge the wake‐up scene (Note S5, Supporting Information). Note that the specific processes of judging scene based on the analysis of the audio signal are introduced in Figure S10, Supporting Information.

When an intrusion occurs, the intrusion detection system will judge the scene: if the intrusion is not initiated by a person, the computer will display “SAFETY;” and if the intrusion is initiated by a person, the wake‐up system will be triggered and the computer will display an “ALARM” alert.

### 2.4 The Power Consumption of the Wake‐Up System

Since the purpose of the N‐ZERO project is to reduce quiescent energy consumption and thereby increasing the battery life, the characteristic of low quiescent power consumption of our system is examined. The wake‐up system consists of a self‐powered bTENG, a switching MOSFET with a leakage current of only 25 nA, and a microcontroller MSP430 with quiescent current consumption of 100 nA in the dormant state according to their data sheets, which is much less than the current micro energy output power (mW level).^[^ ^39^, ^40^ ^]^ Compared with the nondormant system with the bTENG, the wake‐up system with the bTENG that is set to be woke up every 10 min consumes only one‐seventh of the voltage supply, after the same operation time of 140 min. When the wake‐up time interval is set to be 10 min, the voltage supply consumed by the wake‐up system with the bTENG is only half of that consumed by the wake‐up system with a commercial motion/vibration sensor, after the same operation time of 140 min. The wake‐up system with the bTENG has a battery life that is two times longer than the wake‐up system with a commercial motion/vibration sensor; and the battery life of the wake‐up system with the bTENG is 12.4 times longer than that of the nondormant system with a commercial motion/vibration sensor (Figure 3d). These experiments demonstrate that the bTENG‐based wake‐up circuit has ultralow quiescent power consumption, which could meet the requirements of lower power consumption.

### 2.5 The Contact and Noncontact Wake‐Up Modes

To demonstrate the practicability of the bTENG‐based wake‐up system, two types of bTENGs are fabricated to meet the requirements of different wake‐up scenarios: bTENGs shaped like shepherd's purse in the noncontact wake‐up mode and bTENGs shaped like philodendron in the contact wake‐up mode.

**Figure 4:**
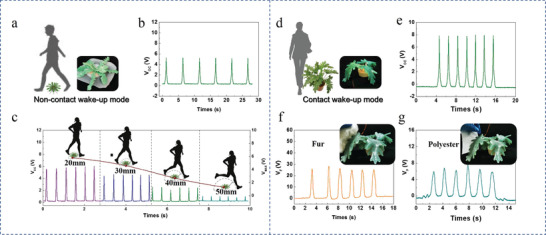
Output characteristics of the bTENG in the noncontact and contact wake‐up modes. a) Schematic diagram of the noncontact wake‐up mode. b) The output voltage of the plant‐shaped bTENG triggered by horizontal contact/separation motion in the noncontact wake‐up mode. c) The relationship between the output voltage and the distance of the noncontact material from the bTENG. d) Schematic diagram of the contact wake‐up mode. e) The output voltage of the plant‐shaped bTENG triggered by horizontal contact/separation motion in the contact wake‐up mode. f) The voltage generated by the bTENG when approached by the fur material. g) The voltage generated by the bTENG when approached by the polyester material.

In the noncontact mode, a bTENG with only the base structure and a bTENG shaped like shepherd's purse structured with a base structure of the same size and seven leaf‐shaped tentacles were fabricated for testing (**Figure** **4**a). The bTENG shaped like shepherd's purse can generate a *V*_oc_ of ≈4.8 V when triggered by noncontact approaching/departing motion, which is 2.4 times higher than the *V*_oc_ of the bTENG with only the base structure (Figure 4b). In addition, the influence of the distance of the noncontact material from the bTENG on the output voltage is studied (Figure 4c). The experimental results show that the bTENG can detect motion within 50 mm, and its output voltage can overcome the threshold voltage of the connected wake‐up circuit, which demonstrates that the bTENG extends the detection range of the wake‐up circuit. In the noncontact mode, the bTENG is connected to a MOSFET with a lower threshold voltage to make it easier to wake up the system. In order to trigger the wake‐up system, the actual output voltage of the bTENG applied on the MOSFET must be higher than the threshold voltage of the MOSFET that is 0.45 V in the noncontact mode.

Since in some application scenarios, the intensity of the wake‐up motion needs to be increased because of the strong environmental interference. We design a taller bTENG to test the output signal in the contact mode. The bTENG shaped like philodendron with a base and four tentacles (Figure 4d) generates a *V*_OC_ of ≈8 V when triggered by slight touches, which is four times higher than the *V*_OC_ of the bTENG with only the base structure (Figure 4e). Both polyester and fur materials can produce high output voltage by slightly touching the bTENG shaped like philodendron (Figure 4f,*g*). In the contact mode, the bTENG is connected to a MOSFET with a higher threshold voltage to avoid false triggering by strong environmental interference. In order to trigger the wake‐up system, the actual output voltage of the bTENG applied on the MOSFET must be higher than the threshold voltage of the MOSFET that is 2 V in the contact mode. The good performance of these two different work modes demonstrates that our bTENG is not limited by appearance and working patterns, which can be changed according to different scenarios.

### 2.6 Demonstrations of the Wake‐Up Modes and Judging the Scene

**Figure 5:**
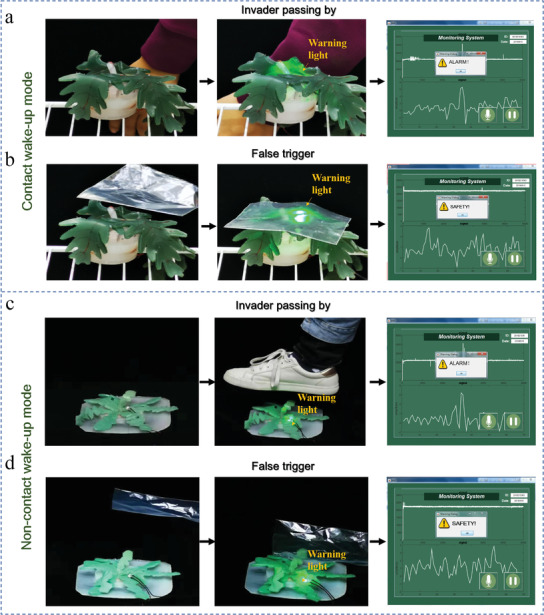
Demonstration and verification of the wake‐up system. a) Photographs show that the intruder touched the bTENG, woke up the circuit, and the computer correctly judged the scene and raised alarm. b) Distinguishing false triggers by the wake‐up system in the contact mode. When a false trigger occurs, for example, a plastic bag contacts the bTENG, the scene is judged to be safe. c) Photographs show that the intruder passed by without touching the bTENG, woke up the circuit, and the computer correctly judged the scene. d) Distinguishing false triggers by the wake‐up system in the noncontact mode. When a false trigger occurs in the noncontact mode, for example, a plastic bag moves above the bTENG, the scene was judged and no alarm was generated.

Verification experiments have been demonstrated by utilizing this wake‐up system to sustainably monitor the safety of the environment, judge the wake‐up scene, and distinguish false triggers in the two types of wake‐up modes. In the experiments, human motion is set as the true trigger and motion of objects is set as the false trigger. In the contact wake‐up mode, when a person passed by the bTENG with contact, this action caused the warning light to illuminate and the remote computer raised alarm based on the analysis of audio signals (**Figure** **5**a; Movie S3, Supporting Information). When a plastic bag fell onto the bTENG, this action also caused the warning light to illuminate, but the remote computer concluded that the condition was safe based on the analysis of audio signals. This indicated that the system successfully woke up the system, judged the scene, and distinguished the intruder (Figure 5b; Movie S4, Supporting Information). Similarly, in the noncontact wake‐up mode, an alarm was generated when a person passed by the bTENG shaped like shepherd's purse without contact, whereas a safety notice was displayed when a plastic bag moved above the bTENG to simulate the false trigger (Figure 5c,d; Movies S5 and S6, Supporting Information). These experiments reveal that our wake‐up system can achieve self‐awakening and scene judgment in both contact and noncontact situations.

## 3 Conclusions

In summary, a bTENG that mimics the plant with lotus‐leaf‐like nanostructures and leaf‐shaped tentacles is developed to serve as a self‐powered motion sensor in the wake‐up circuit with the property of capturing slight external mechanical disturbances. The bTENG with leaf‐shaped tentacle structures on the base can increase the voltage output by four times compared with the TENG with only the base. The systematic optimization of tentacle length, tentacle number, silicone rubber thickness, and metal wire diameter is also investigated to prove the superiority of the leaf‐shaped tentacle. The wake‐up system based on the bTENG has an increased detection range towards slight external mechanical disturbances and extended distance range, which can work in both the contact and noncontact modes. A wake‐up circuit is specially designed to be compatible with the bTENG, which integrates a switch component that can more accurately control the wake‐up system. Moreover, an intrusion detection system based on audio recognition of motion has also been established to judge the scene and distinguish human motion. Our work has the characteristics of low quiescent power consumption (125 nA theoretical quiescent current consumption), large wake‐up range that can be awakened by human striding over or slight touch, low cost, and environmental concealment. This work overcomes a bottleneck problem of the power limitations of persistent sensing and provides a new wake‐up method with low quiescent power consumption, which can be widely applied in the areas such as Internet of things and remote environmental monitoring.

## 4 Experimental Section

##### Fabrication of Triboelectric Nanogenerators

Polydimethylsiloxane (PDMS) mixed in a ratio of 1:5 (DC184) was poured onto the positive surface of the dried lotus leaf cut in a leaf shape. The solution stood for half an hour to expel bubbles, and then was heated for full solidification (25 °C, 25 min). After the solidification of PDMS, the dried lotus leaves were removed from the surface of the PDMS. Copper wire wound in vein shape and silicone rubber (Ecoflex 00‐30) mixed in 1:1 ratio was put into PDMS mold. The wire at the tail of the tentacle was exposed and connected with a metal plate. The metal plate connected with the blade‐shaped component was placed on a Petri dish that had solidified a layer of silicone rubber beforehand, and then the silicone rubber was poured into the Petri dish for solidification. After curing, the silicone rubber and PDMS could be separated. The device could be folded in half to break the PDMS without damaging the silicone rubber, so as to separate the silicone rubber and the PDMS.

##### Electrical Measurements

The output *V*_OC_, *I*_SC_, and *Q*_SC_ of the TENG were measured by a voltage preamplifier (Keithley 6514). One terminal of the instrument was connected with the output of the TENG, and the other terminal was connected to the ground. The software platform was constructed based on LabVIEW, which was capable of realizing real‐time data acquisition control and analysis. A field emission scanning electron microscope (GeminiSEM 500, ZEISS) was used to characterize the surface morphologies of the etched film.

## Conflict of Interest

The authors declare no conflict of interest.

## Supporting information

Supporting Information

Supplemental Movie 1

Supplemental Movie 2

Supplemental Movie 3

Supplemental Movie 4

Supplemental Movie 5

Supplemental Movie 5
