# Supplementary material for: Ultralow Quiescent Power‐Consumption Wake‐Up Technology Based on the Bionic Triboelectric Nanogenerator
Source: Adv Sci (Weinh). 2020 May 11;7(12):2000254. doi: 10.1002/advs.202000254 (PMC7312437; doi:10.1002/advs.202000254)
Supplement: Supplementary file 1 — Supporting Information [file ADVS-7-2000254-s001.pdf]

Supporting Information

Ultra-low Quiescent Power-Consumption Wake-up Technology Based on the Bionic Triboelectric Nanogenerator

Chenxi Zhang, Keren Dai, Di Liu, Fang Yi, Xiaofeng Wang*, Lianqing Zhu and Zheng You*

C. Zhang
Department of Precision Instrument, Tsinghua University, Beijing, 100084, P. R. China
Prof. X. Wang, Prof. Z. You
Beijing Innovation Center for Future Chips, Tsinghua University, Beijing, 100084, P. R. China
Center for Flexible Electronics Technology, Tsinghua University, Beijing 100084, P. R. China
Department of Precision Instrument, Tsinghua University, Beijing, 100084, P. R. China
E-mail: xfw@mail.tsinghua.edu.cn (Xiaofeng Wang)
Prof. F. Yi
School of Materials Science and Engineering, Sun Yat-sen University, Guangzhou 510275, Guangdong, P.R. China
E-mail: yifang@mail.sysu.edu.cn (Fang Yi)
Prof. K. Dai
ZNDY of Ministerial Key Laboratory, School of Mechanical Engineering, Nanjing University of Science and Technology, Nanjing 210094, P. R. China
D. Liu
Beijing Institute of Nanoenergy and Nanosystems, Chinese Academy of Sciences, Beijing 100083, P. R. China.
Prof. L. Zhu
School of Instrument Science and Opto-Electronic Engineering, Beijing Information Science and Technology University, Beijing 100192, P. R. China

Figure S1. The fabrication processes of the leaf-shaped tentacles of the bTENG.

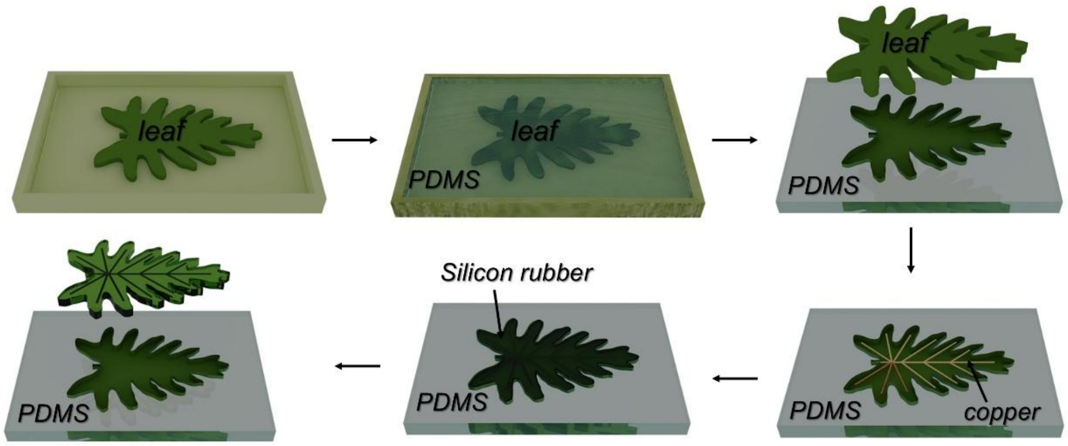

Figure S2. The relationship between the output voltage of the TENG and the exerted pressure in contact mode. (It can be obtained that the pressure sensitivity is 34.4 mV/Pa when the pressure is less than 160 Pa and the pressure sensitivity is 8.3 mV/Pa when the pressure is higher than 160 Pa.)

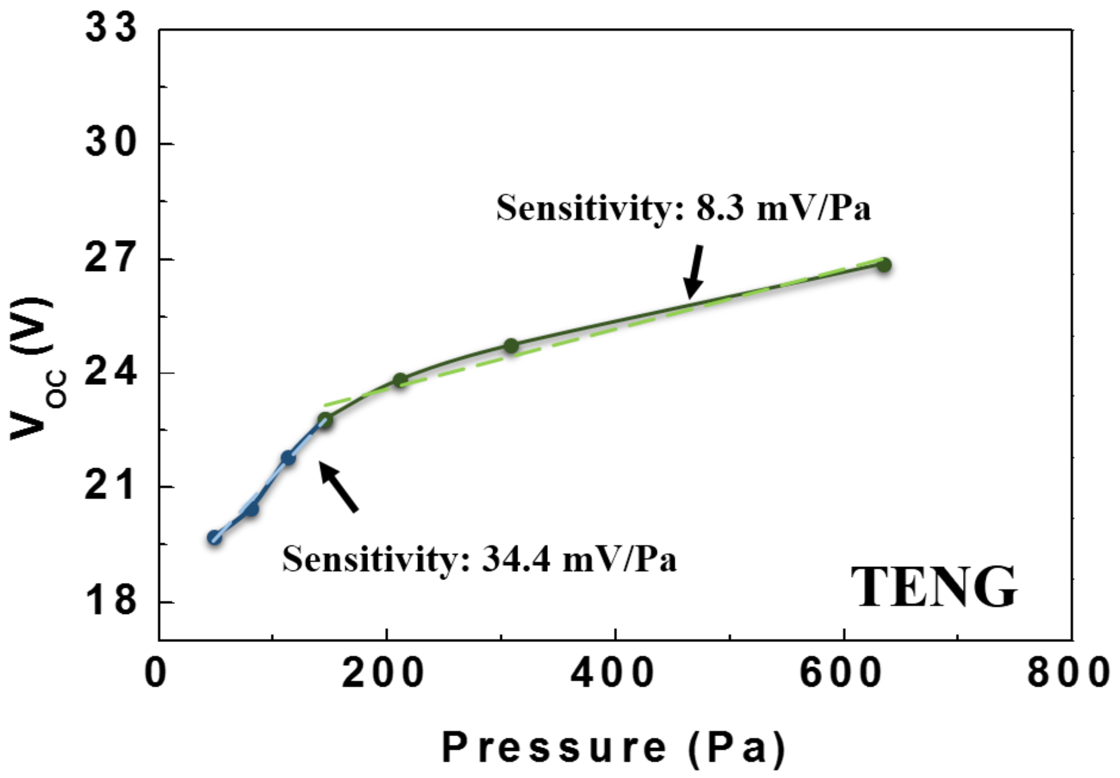

Figure S3. The relationship between the voltage and the pressure when objects of different materials exerted pressure on the TENG.

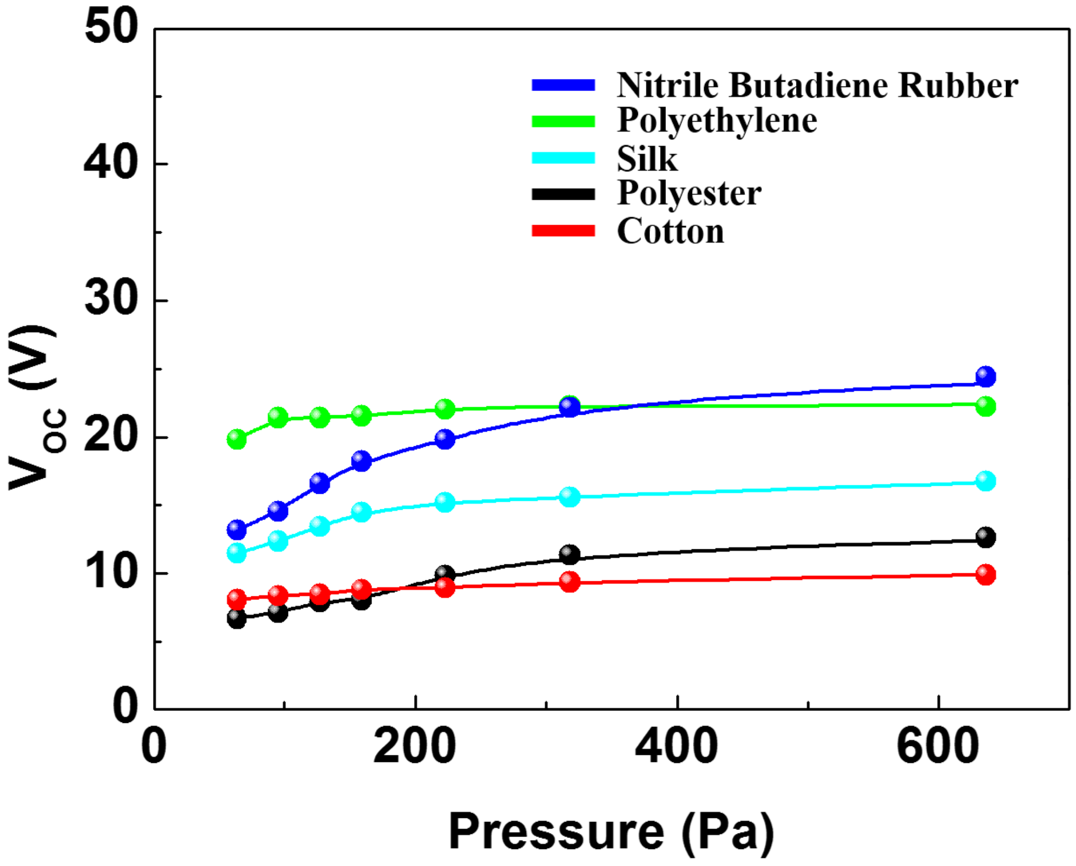

Figure S4. a) The relationship between the output voltage of the PENG and the nearest distance from the approaching material in the non-contact experiment. The inset shows the output voltage of the PENG when the nearest distance is 4 mm. b) The relationship between output voltage of the PENG and the exerted pressure during contact. The inset shows the output voltage signals of the PENG when exerted a pressure of 95 Pa. c) The simulation results of the relationships between the output voltages and the nearest distances in the non-contact mode for the TENG and PENG. d) The output voltage signals of the PENG in the feather experiment.

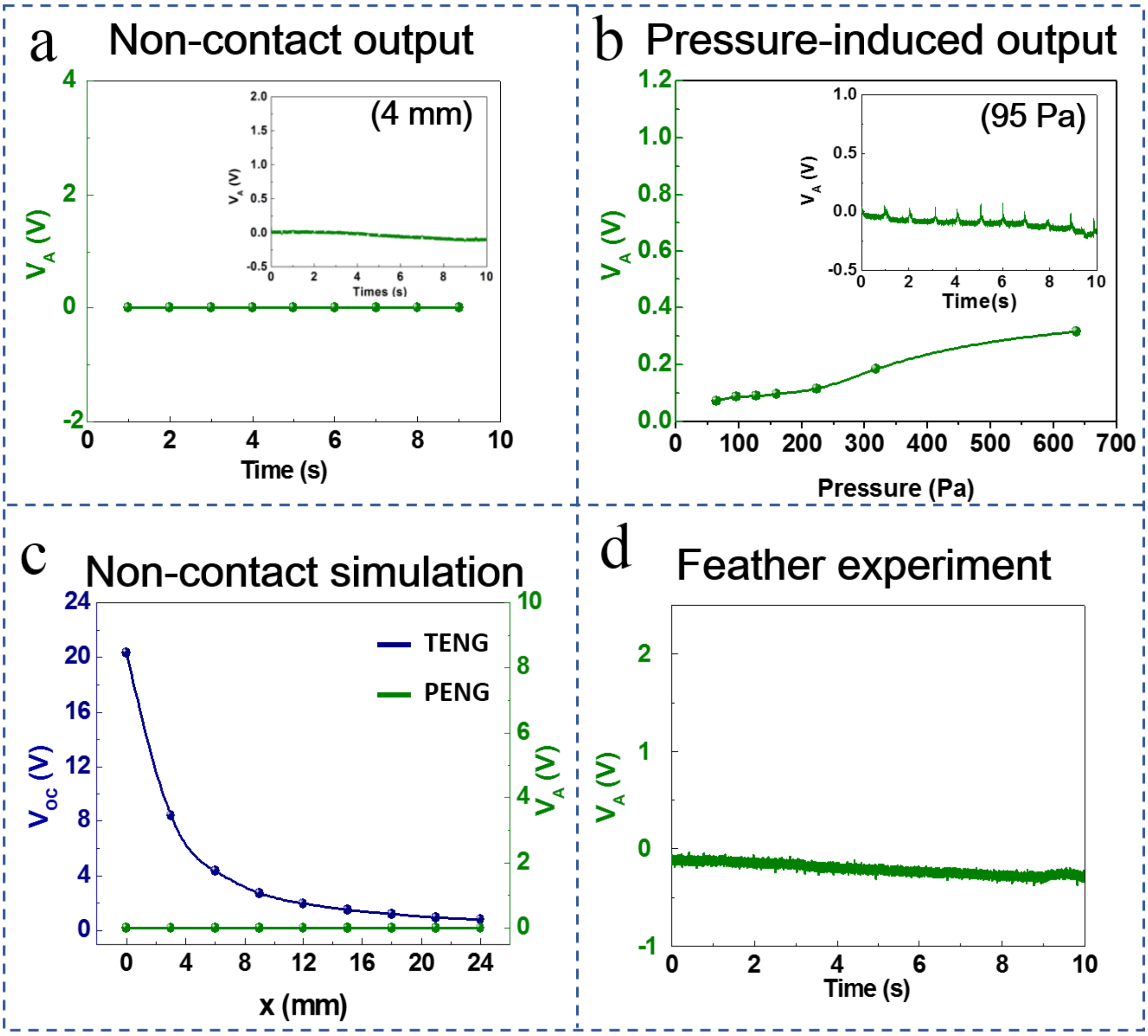

Figure S5 a) Schematic diagrams depicting the simplified models of the TENG and the PENG. b) The equivalent circuits of the TENG and PENG. Note that the detailed explanation for the simulations can be found in Supporting Note S1.

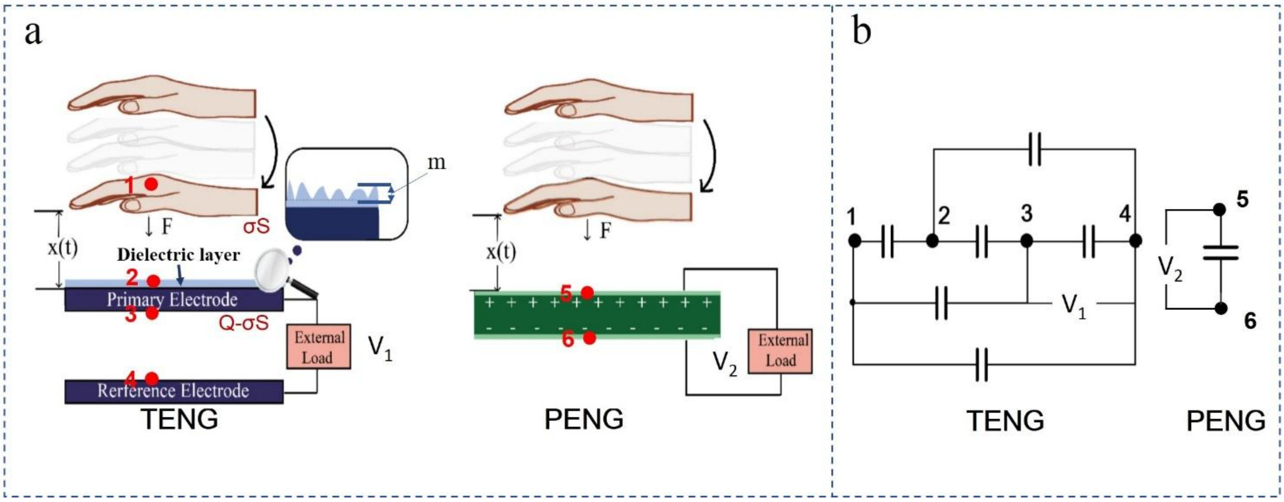

Figure S6. a) Photographs of the flat TENG with the base structure and the simplified TENG with cylindrical tentacles. Note that n is the number of the tentacle, L is the length of the tentacle, D is the layer thickness of the external silicone rubber, d is the radius of the internal wire. b) Simulation results of the operation mode for the TENG with tentacles. c) The simulated potential change of the working electrode when the contact material works in reciprocating motion.

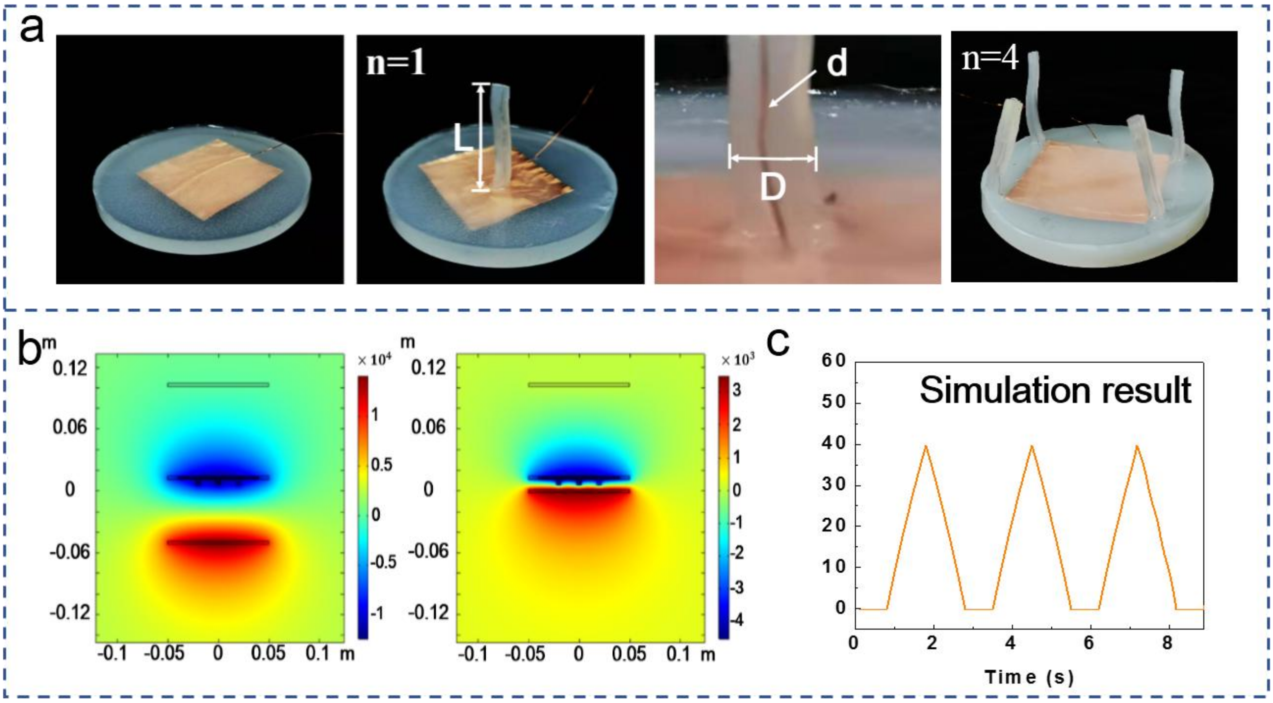

Figure S7. The simulation results of the effects of parameters of device structure on the electrical outputs. a) The relationship between the output voltage of the TENG and the number of tentacles. b) The relationship between the output voltage and the length of tentacles. c) The relationship between the output voltage and the layer thickness of the external silicone rubber tentacles. d) The relationship between the output voltage and the radius of the internal wire tentacles.

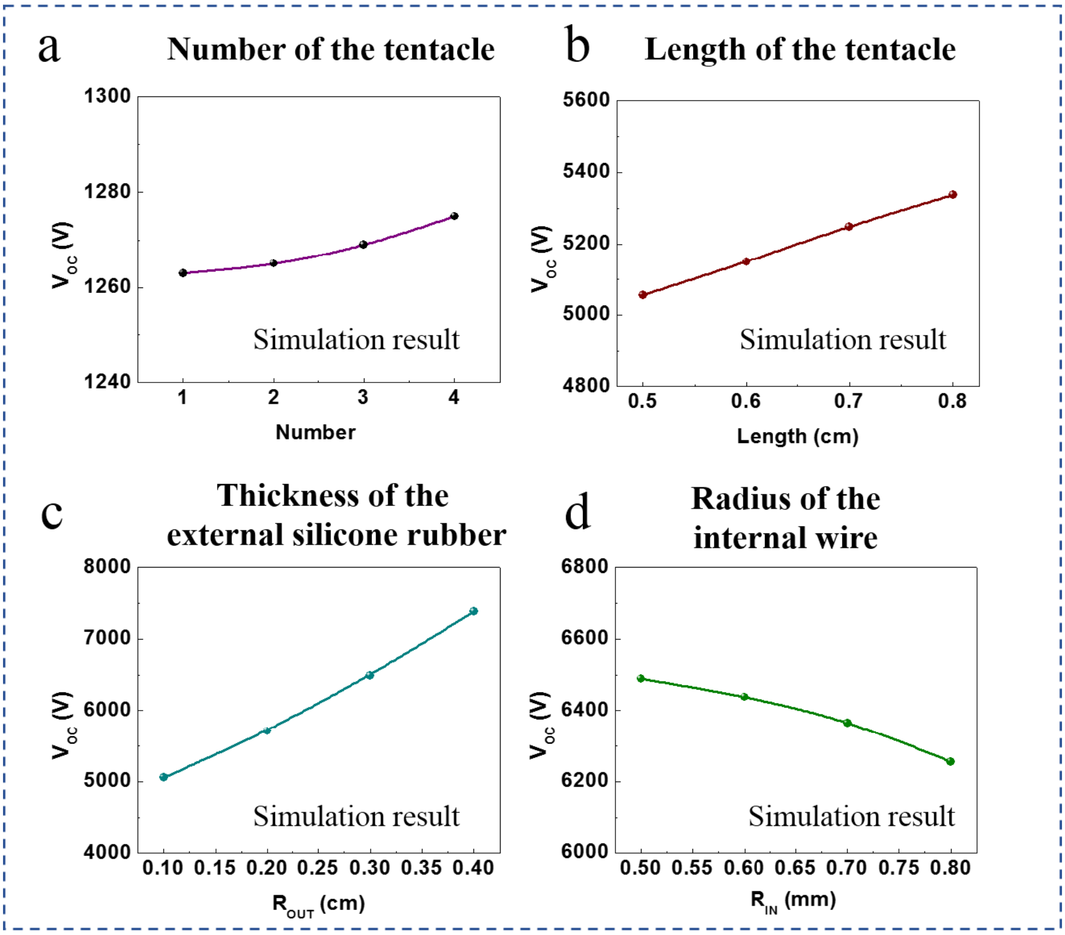

Figure S8. The simulation results about the influence of the tentacle distribution on the output voltage.

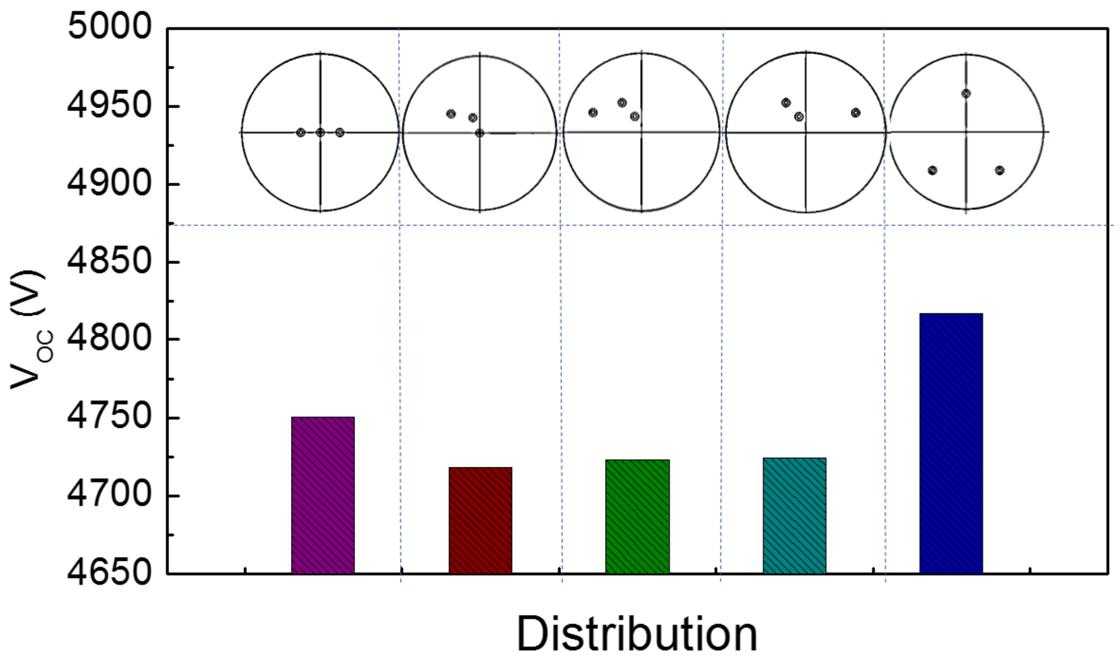

Figure S9. The impacts of the parasitic capacitance and threshold voltage of the MOSFET on the wake-up system. a) The output voltage of the MOSFET PMZ600UNEL. b) The output voltage of the MOSFET IRF1404. c-d) The actual voltages of the bTENG applied to the c) MOSFET PMZ600UNEL and d) MOSFET IRF1404, respectively. e-f) The open-circuit voltages of the bTENG integrated with e) MOSFET PMZ600UNEL and f) MOSFET IRF1404, respectively. Note that the parasitic capacitance of the PMZ600UNEL is 18.3 pF, the parasitic capacitance of the IRF1404 is 6862 pF; the threshold voltage of the PMZ600UNEL is 0.45 V, the threshold voltage of the IRF1404 is 2 V. Both of the output voltages of the two MOSFETs wake up the system successfully.

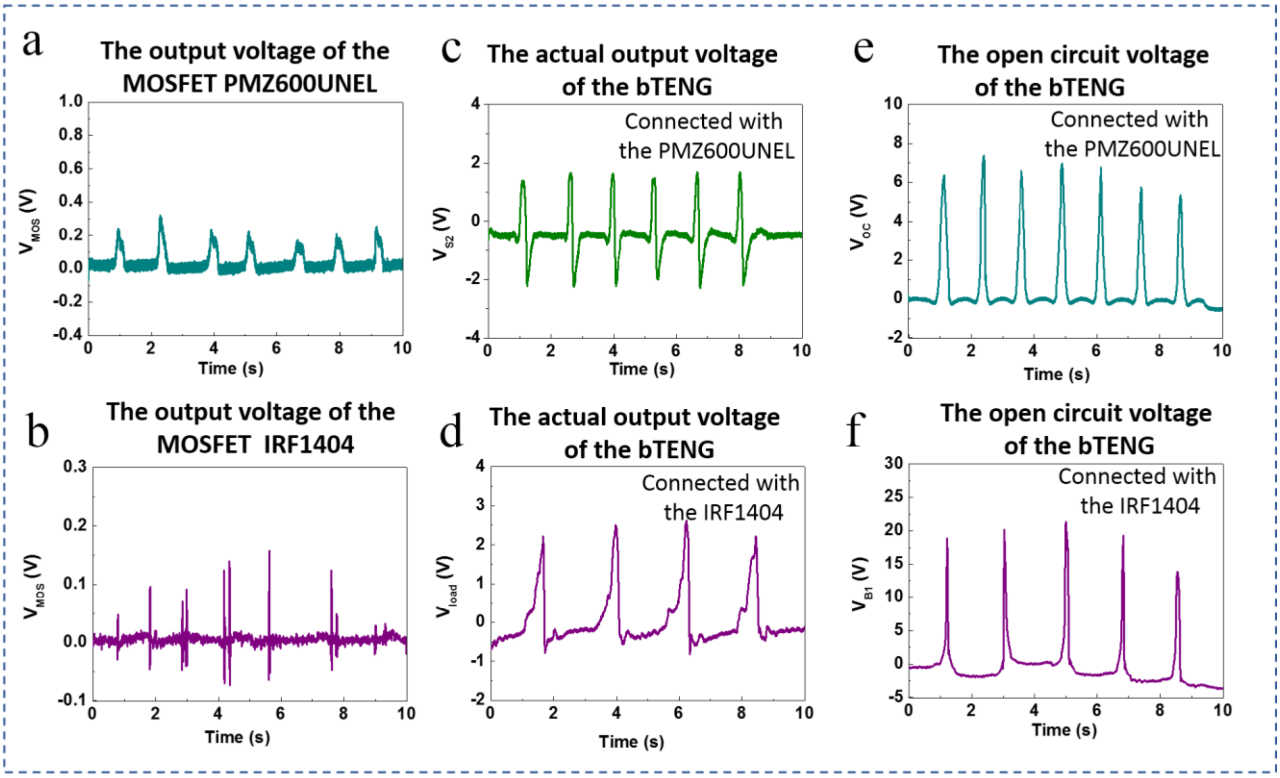

Figure S10. The process of judging scene based on the analysis of the audio signal.

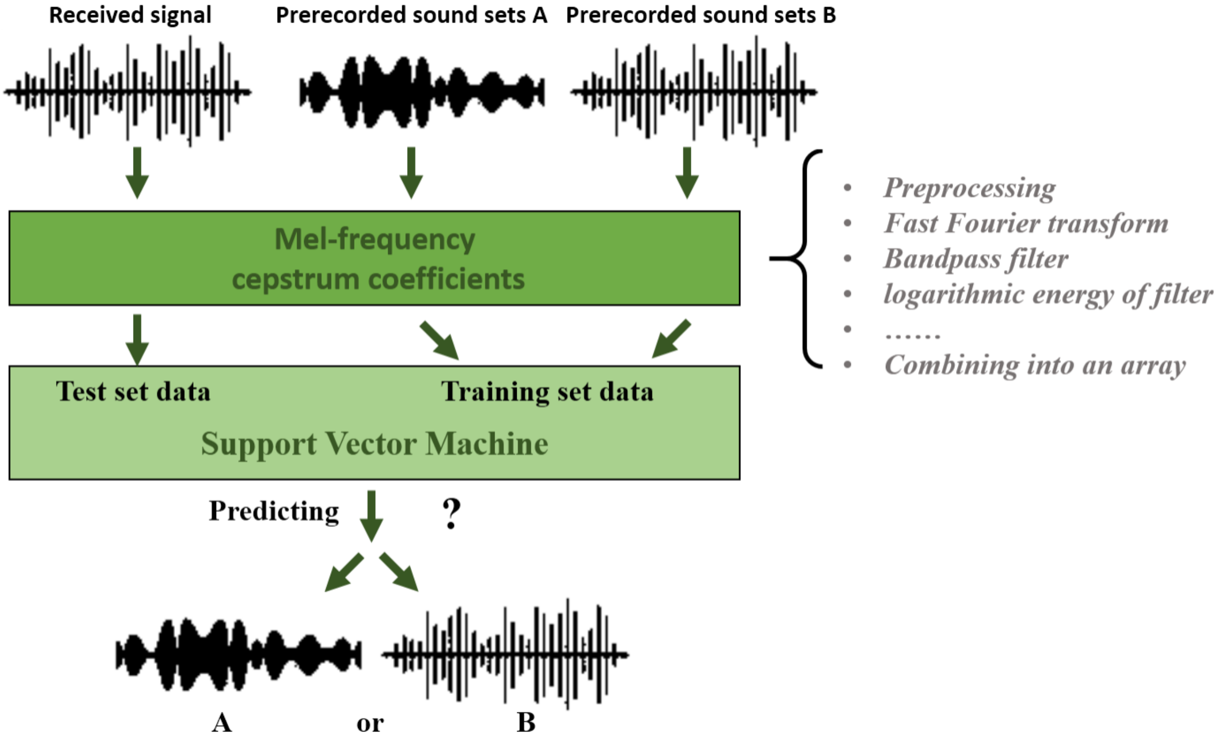

Supporting Note S1：Theoretical analysis of the TENG and the PENG for detecting slight touch

The simulation results of the relationship between the output voltages and the nearest distance from the approaching material in the non-contact mode for the TENG and PENG consist with the experimental results (Figure S4c).

We simplify the TENG and the PENG into models as shown in Figure S5a, in which x represents the nearest distance from the approaching material. In the TENG model, m represents the height of the non-smooth region, two processes mentioned above can be considered to be $x < m$ and $x > m$. The TENG is structured with a copper plate wrapped by silicon rubber layer. One terminal of the instrument is connected with the copper plate of the TENG, and the other terminal is connected to the ground. In the simplified model, the palm represents the approaching object, the primary electron represents the copper plate inside the TENG, the reference electron represents the ground connected with the experimental instrument and the external load represents the measuring instrument. The light blue area above the primary electron is a dielectric area indicating the silicon rubber material used to increase friction. In the PENG model, the dark green region represents the film with piezoelectric properties, and the light color regions are the electrodes connected with the piezoelectric film.

Our TENG is working in dielectric-to-dielectric contact-separation mode. The equivalent circuit is shown in Figure S5b, where V_1 represents the output voltage of the TENG and ports 1,2,3,4 represent the approaching object, the dielectric upper surface, the primary electron and the reference electron of the TENG respectively. According to the previous research, the TENG with dielectric-to-dielectric contact-separation mode can output high voltage when the external object approaches the TENG. [27] The PENG can be simplified as an equivalent capacitance, its output voltage V_2 is observable only when the applied pressure is large enough.

From a physical point of view, both experiments are the process that the external object

approaches the friction layer gradually, which is essentially the same. The output voltage of the TENG is due to the changing position of the charged surface, so there is also a voltage output when the object is not in full contact with the TENG. As for the PENG, these two experiments are totally two different cases: generating and not generating strain. The PENG's output voltage is positively correlated with the strain, so there is no available output voltage under slight contact conditions that do not induce strain. It could be said that TENGs are more suitable as self-powered sensors for light touch detections.

Supporting Note S2：The selection of the MOSFET.

The output characteristics of the TENG are: high voltage output and low current output, and the output voltage of the TENG will be affected by the load impedance. Hence, the wake-up system can be more accurately controlled by adding a MOSFET with a known impedance after the bTENG. In addition, the MOSFET has the characteristics of easy conduction, no restriction on the input current and low power consumption. Therefore, we connect the bTENG to the gate electrode of the MOSFET as a switch element. In a conventional MOSFET, the gate electrode G, source electrode S, and drain electrode D can control its output performance. The gate voltage V_{GS} controls the charge carrier channel between the source and drain electrodes. When the input V_{GS} is higher than the threshold voltage of the MOSFET, the switch will turn on and give a trigger voltage to the microcontroller to start the whole circuit, which gives us the enlightenment that we can control the wake-up voltage of the wake-up system by selecting the threshold voltage of the MOSFET. The previous theoretical analysis shows that the actual output voltage of the TENG will be increased with the decrease of the capacitance value of the connected load that when the open-circuit voltages of the bTENG are the same.[20] Choosing the MOSFET with a small parasitic capacitance is beneficial to enlarge the wake-up range of the system.

The capacitance parameters listed in the MOSFET's datasheet are input capacitance C_{iss}, output capacitance C_{oss} and reverse transmission capacitance C_{rss}. The relationship among them are:

$$\begin{aligned} &C_{iss} = C_{GS} + C_{GD} \\ &C_{oss} = C_{DS} + C_{GD} \\ &C_{rss} = C_{GD} \\ &\therefore C_{GS} = C_{iss} - C_{rss}, C_{DS} = C_{oss} - C_{rss} \end{aligned} \tag{1}$$

Therefore, the MOSFET with small difference between the input capacitance and the reverse transmission capacitance is selected in this experiment. In order to verify the above conclusions about

parasitic capacitance, we choose two different types of MOSFET for the experiment, PMZ600UNEL with the parasitic capacitance of 18.3 pF and IRF1404 with the parasitic capacitance of 6862 pF. When repeating the same mechanical motion, the open circuit output voltage of the bTENG, the actual output voltage of the bTENG connected with a MOSFET and the output of the MOSFET are recorded. In Figure S9, the green curve is the output signal of the system with the PMZ600UNEL, and the purple curve is the output signal with the IRF1404. The output signals of these two MOSFETs show that both of them wake up the system successfully (Figure S9a, b). The actual output voltage amplitude of these MOSFETs is basically the same. Note that the actual voltages of the bTENG on the two MOSFETs are maintained the same (Figure S9c, d). And when the actual voltages of the bTENGs applied to the two MOSFETs are the same, the open-circuit voltage of the bTENG connected with the MOSFET of a higher parasitic capacitance (IRF1404) is 3 times higher than that connected with the MOSFET of a lower parasitic capacitance (PMZ600UNEL) (Figure S9e, f). This proves that when the open-circuit voltages of the bTENG are the same, the smaller the MOSFET’s parasitic capacitance, the larger the actual output voltage of the bTENG applied to the MOSFET. It is also found that when the actual voltages of the bTENGs applied to these two MOSFETs are the same, the MOSFET of a smaller threshold voltage can output higher electrical signals, which indicates that the bTENG with the MOSFET of a smaller threshold voltage can wake-up the system more easily (Figure S9a, b).

Therefore, the MOSFETs with low threshold voltages and small parasitic capacitances are selected in this paper to enlarge the wake-up range. At the same time, the MOSFET needs to have the characteristics of low drain leakage current to reduce the power consumption of the system. If other types of TENGs with really high voltage output are connected with the MOSFET, the breakdown voltage of the MOSFET should also be considered.

Supporting Note S3：The basic description of MSP430 and the core program of hardware

The Texas Instruments MSP430 family of ultralow-power microcontrollers consist of several devices featuring different sets of peripherals targeted for various applications. The architecture, combined with five low power modes is optimized to achieve extended battery life in portable measurement applications. The device features a powerful 16-bit RISC CPU, 16-bit registers, and constant generators that attribute to a maximum code efficiency. The digitally controlled oscillator (DCO) allows wake-up from low-power modes to active mode in less than 6μs.The MSP430x13x and the MSP430x14x series are microcontroller with two built-in 16-bittimers, a fast 12-bit A/D converter, one or two universal serial synchronous/asynchronous communication interfaces (USART), and 48 I/O pins.

Supporting Note S4： Extract the Mel-frequency cepstrum coefficients (MFCC) of the recorded signal

In the field of speech processing, Mel frequency cepstrum (MFC) represents the short-term power spectrum of speech, which is the linear cosine conversion result of logarithmic power spectrum on the nonlinear mel scale of frequency. We preprocess the speech signal into a short MFCC which can fully express the content. This is conducive to reducing the dimension size of support vector machine, and can greatly reduce the amount of calculation while ensuring accuracy.

Extraction steps:

1. High-pass filter
2. Framing
3. Windowing
4. Fast Fourier transform
5. Bandpass filter
6. Calculating logarithmic energy of filter bank output
7. Discrete Cosine Transform
8. Calculating logarithmic energy per frame
9. Extraction of Dynamic Difference Parameters

Key program:

1. bank=melbankm(…);
2. dctcoef(…,:)=cos((2*n+1)*k*pi/(2*24));
3. xx=filter(…,1,xx); xx=enframe(xx,…);
4. c1=dctcoef*log(bank*…);
5. dtm(i,:)=-2*m(i-2,:)-m(i-1,:)+m(i+1,:)+2*m(i+2,:);

6. dtmm(i,:)=-2*dtm(i-2,:)-dtm(i-1,:)+dtm(i+1,:)+2*dtm(i+2,:);

Supporting Note S5：Machine learning using support vector machine

Support Vector Machine (SVM) is a binary classification model. Its purpose is to find a hyper plane to segment samples. The principle of segmentation is to maximize the interval, and finally to solve a convex quadratic programming problem. Because the conditions of our experiment are relatively simple, we only discuss the linear separable case. By maximizing the hard interval, we can learn a linear branching support vector machine. Given the training sample set, the basic idea of classification learning is to find a partitioning hyper plane in the sample space based on the training set, and separate different types of samples. If a linear function can separate samples, these data samples are said to be linear separable. It seems intuitively that there are many hyper planes that can separate training samples, and the optimal division corresponds to the hyper planes that can correctly divide the data and have the largest interval. In the training process, the computer can accurately divide the hyper plane by calculating the existing training set. In the process of prediction, the computer will compare the predicted data with the hyper plane, and judge the corresponding situation according to the position relationship between the data and the hyper plane.

Key program:

```
SVMStruct = svmtrain(a,label,'Showplot',true);
Group = svmclassify(SVMStruct,mfcc_array,'Showplot',true);
if(Group==0)
      h=warndlg('ALARM！ ');
     ha=get(h,'children');
     hu=findall(allchild(h),'style','pushbutton');
     set(hu,'string','ok');
     ht=findall(ha,'type','text');
     set(ht,'fontsize',20 ');
else
```

```
  h=warndlg('SAFETY!');
 ha=get(h,'children');
 hu=findall(allchild(h),'style','pushbutton');
 set(hu,'string','ok');
 ht=findall(ha,'type','text');
 set(ht,'fontsize',20,);
end
```
